# The Design and Optimization of a Highly Sensitive and Overload-Resistant Piezoresistive Pressure Sensor

**DOI:** 10.3390/s16030348

**Published:** 2016-03-09

**Authors:** Xiawei Meng, Yulong Zhao

**Affiliations:** The State Key Laboratory for Manufacturing Systems Engineering, Xi’an Jiaotong University, Yan Xiang Road, Xi’an 710049, China; summer_meng@stu.xjtu.edu.cn

**Keywords:** micro-pressure, piezoresistive sensor, high sensitivity, high overload

## Abstract

A piezoresistive pressure sensor with a beam-membrane-dual-island structure is developed for micro-pressure monitoring in the field of aviation, which requires great sensitivity and overload resistance capacity. The design, fabrication, and test of the sensor are presented in this paper. By analyzing the stress distribution of sensitive elements using the finite element method, a novel structure incorporating sensitive beams with a traditional bossed diaphragm is built up. The proposed structure proved to be advantageous in terms of high sensitivity and high overload resistance compared with the conventional bossed diaphragm and flat diaphragm structures. Curve fittings of surface stress and deflection based on ANSYS simulation results are performed to establish the sensor equations. Fabricated on an *n*-type single crystal silicon wafer, the sensor chips are wire-bonded to a printed circuit board (PCB) and packaged for experiments. The static and dynamic characteristics are tested and discussed. Experimental results show that the sensor has a sensitivity as high as 17.339 μV/V/Pa in the range of 500 Pa at room temperature, and a high overload resistance of 200 times overpressure. Due to the excellent performance, the sensor can be applied in measuring micro-pressure lower than 500 Pa.

## 1. Introduction

Most equipment requires the use of sensor technology to monitor its working status in terms of collecting spindle vibration, cutting force, working temperatures [1,2,3,4,5], *etc.* In particular, with the development of aviation engineering technology a number of piezoresistive pressure sensors are desired for micro-pressure measurements [6,7,8,9].

In the field of aviation, the altitude location of a vehicle can be determined through the measurement of pressure, due to the relationship between pressure and height. Therefore, high sensitivity is the most important factor for any employed pressure sensor to ensure the accuracy of orbital correction of a vehicle in the upper air. Besides, high overload resistance is also needed in case of complicated and severe weather conditions. Apparently, a pressure sensor with high sensitivity and strong anti-overload ability is necessary for the rapid development of the aviation field.

Since the first silicon piezoresistive pressure sensor was developed in 1962 [10], MEMS pressure sensors have been widely used in various industries, stimulated by the advantages offered by bulk and surface micromachining techniques, low power consumption and miniaturization. Because of its excellent linearity and fine sensitivity as well as simple and direct signal transduction mechanism between the mechanical and the electrical domains, the pressure sensor based on MEMS has satisfied the demand of the aviation field.

There have been diverse configurations since the piezoresistive pressure sensor was put into wide use. The pressure sensor’s performance was improved by eliminating the bonding and replacing the metal diaphragm with single crystal silicon for the first time [10]. The performances were further improved by using piezoresistive pressure sensors. Then Kurtz *et al.* [11] developed a micro-pressure sensor based on the bossed diaphragm structure featuring relatively better sensitivity and linearity. After the 1980s, continued improvements in sensing configuration design led to further reductions in size, increases sensitivity, higher yield, and better performance. Shimazoe and Matsuoka [12] developed a sensor with a center boss on the diaphragm and an annular groove formed on the back surface of the diaphragm. Although the accuracy of the sensor was 0.17% full scale (FS), the variation of stress distribution was evident, and thus the high precision placement of piezoresistors was demanded to ensure the piezoresistors were in the appropriate locations. Moreover, the sensor was unfavorable to miniaturization and batch production. Bao *et al.* [13] proposed a beam-diaphragm structure by introducing beams on the flat membrane of twin isles structure and forming a shape like a dumbbell. The sensitivity was 0.699 μV/V/Pa, slightly lower for the operating range of 1 kPa, although the overload resistance was enhanced to 140 times overpressure and the nonlinearity of 0.25% FS was satisfactory. Johnson *et al.* [14] reported a novel ribbed and bossed structure. The incorporation of ribs into the diaphragm for stress concentration proved effective in enhancing the sensitivity and reducing deflection. Additionally, the introduction of a self-aligning rim facilitated manufacture. However, the overload resistance was not high enough for the field of aviation due to its thin bosses. Tian *et al.* [1] designed a beam-membrane structure through etching the cross beam on the flat diaphragm and the stiffness was increased, resulting in a satisfactory linearity (the nonlinearity was 0.09% FS) for measurements of 5 kPa. Nevertheless, the overload resistance as well as the sensitivity of 1.549 μV/V/Pa was relatively low for use in the aviation field. With a high sensitivity micro-pressure sensor, the altitude of even one stair can be sensed [6]. To detect altitude variations of less than 100 m, the target pressure range is set as 500 Pa, and both the high sensitivity and a high overload capacity beyond 200 times measurement are required. Simultaneously, a feasible fabrication process is also expected for mass production. Unfortunately, most of the sensors mentioned above are not able to fully meet all these requirements.

Now that the structures discussed above are not satisfactory for micro-pressure measurements, a novel beams-membrane-dual-island structure originated from the bossed diaphragm is put forward. By incorporating beams into the diaphragm, stresses are expected to be concentrated. In addition, high overload resistance is anticipated due to the existence of islands to limit the displacement. As the silicon bulk micromachining is utilized to realize the proposed sensor, high yield and low cost can be expected. To verify the scheme, a finite element method (FEM) model, nonlinear optimization, and experiments are implemented.

## 2. Experimental Section

### 2.1. Structural Design

**Figure 1:**
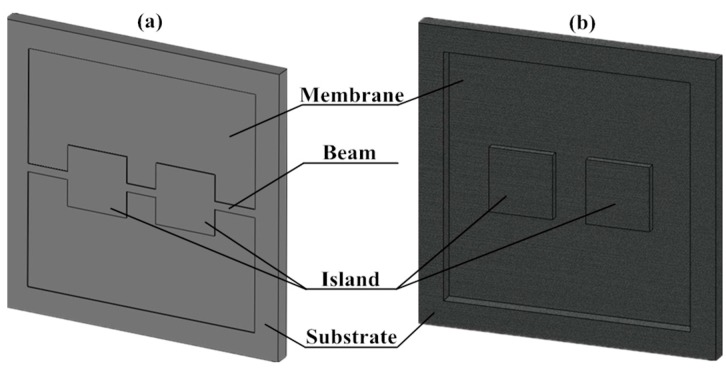
(**a**) The schematic diagram of the proposed structure in the front view; (**b**) The rearview of the structure without bonding glass.

**Figure 2:**
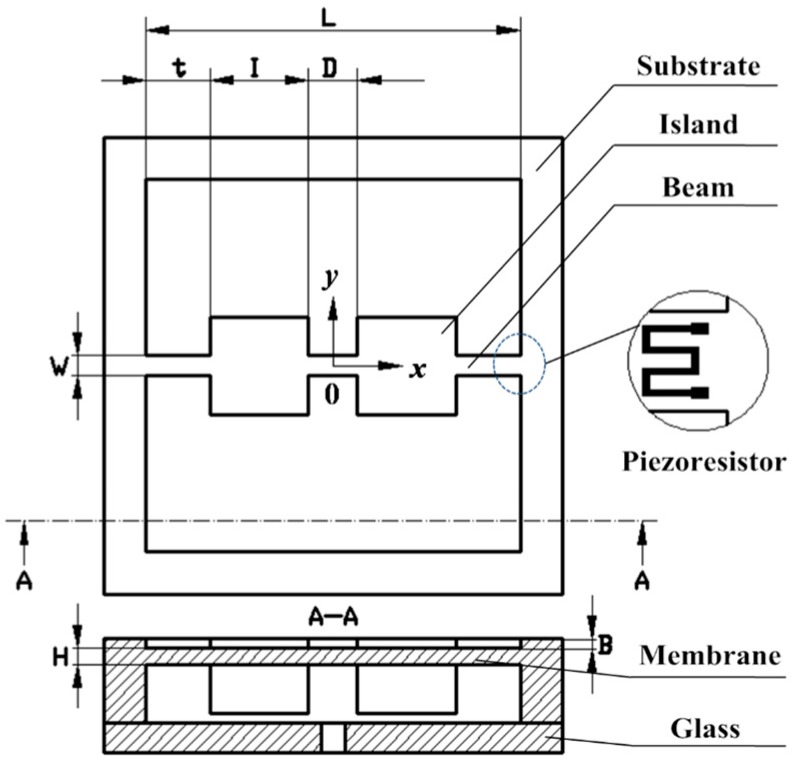
The front and cross-sectional view of the proposed structure.

To protect the silicon structure from being fragmentized under the atmosphere pressure, 200 times higher than the operating range, a bossed diaphragm is adopted as shown in Figure 1. With the support of mass, the membrane could withstand the high overload of 200 times overpressure without being broken. What’s more, the introduction of mass improves the stiffness of the structure, which results in an acceptable linearity. Simultaneously, the improvement of the stiffness sacrifices the effective stress which determines sensitivity. To satisfy the demands for high sensitivity and overload resistance, a novel structure based on bulk silicon process is constructed as shown in Figure 2, where *L* is the effective width of the membrane, *I* is the top width of the island, *D* is the distance between two islands, *t* is the distance between an island and the side of the membrane, and *W* is the beam’s width, respectively. *H* and *B* are the thicknesses of the membrane and beams, respectively. In this figure, one of the piezoresistors arranged on the beams is enlarged. Besides, the bonding glass on the backside can be observed clearly from the cross-sectional view. In the configuration, two islands are connected by three beams located on the membrane. The dorsal cavity provides the space for deformation. The structure is designed for 500 Pa micro-pressure measurements.

### 2.2. Structural Analysis

The ranges of all the variables listed in Table 1 are constrained by the level of processing technology, certain scopes, actual demands and reliability. To obtain the optimal dimensions of the sensor structure, the optimization model is constructed [15]:

(1) $$\max(\sigma_{\mathrm{eqv}})$$

(2) $$\omega_{\max}\leq 0.2H$$

(3) $$\sigma_{\mathrm{overload}}\leq \frac{\sigma_{b}}{n}$$

(4) $$\sigma_{b}\leq 7\mathrm{GPa}$$

where *σ*_eqv_, *ω*_max_, *σ*_overload_, *σ*_b_ and *n* are the maximum Mises stress, maximum deflection, maximum overload pressure (100 kPa), ultimate strength of ingle crystal silicon, and safety factor (*n* = 11).

**Figure 3:**
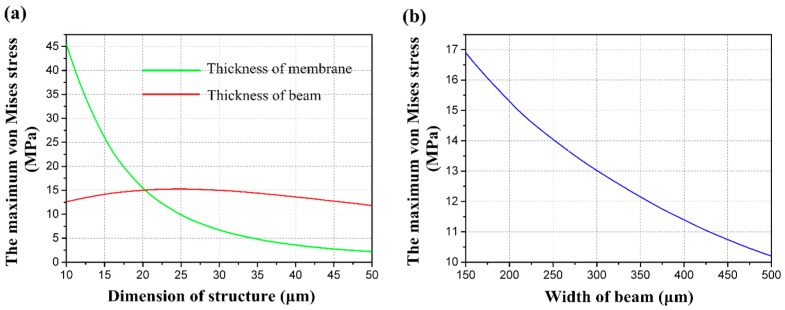
(**a**) The relationship between the maximum von Mises stress and dimensions of the structure; (**b**) The relationship between the maximum von Mises stress and beam width.

**Figure 4:**
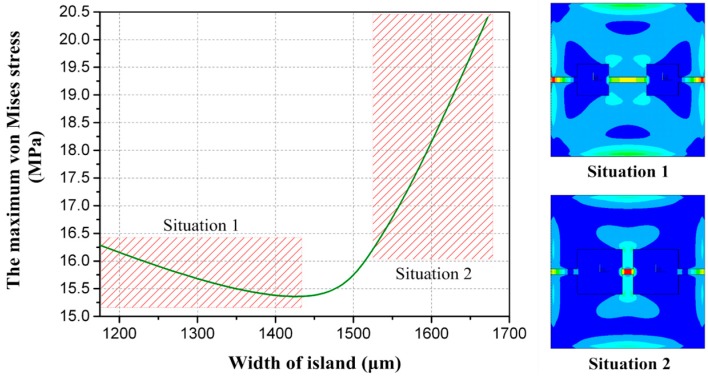
The relationship between the maximum von Mises stress and width of an island.

To determine the structural dimensions, the relationship between single structure dimensions and the maximum von Mises stress has been researched. The influences of structure dimension variables on the maximum von Mises stress *σ*_eqv_ are plotted in Figure 3. Descriptions of the dimension of the proposed structure are as follows: it is obvious that membrane thickness *H* has a great effect on the maximum von Mises stress. The beam thickness *B* and the beam width *W* are secondary. On the other hand, when the membrane width *L* is determined, island width *I* and beam length *t* also affect the maximum von Mises stress. On account of the locations where the four resistors are arranged, determination of both variables should refer to the stress distribution of the structure. The uniformity of the distribution needs to be taken into consideration as well as the maximum von Mises stress. As Figure 4 shows, the two situations are supposed to be avoided. The relationship between beam length *t* and the maximum von Mises stress is extremely similar to that of island width *I*, and the influence of beam length *t* on maximum von Mises stress is small, and can be ignored.

According to the influences of the structural dimension variables on the maximum von Mises stress and the optimization model, the dimensions of the proposed structure are finally determined. The overall dimensions of the sensor die are 7000 μm × 7000 μm, with a 20 μm thickness membrane, a 1500 μm width island, two 25 μm thickness and 980 μm width beams, and another 750 μm width beam to connect two islands in the middle.

**Figure 5:**
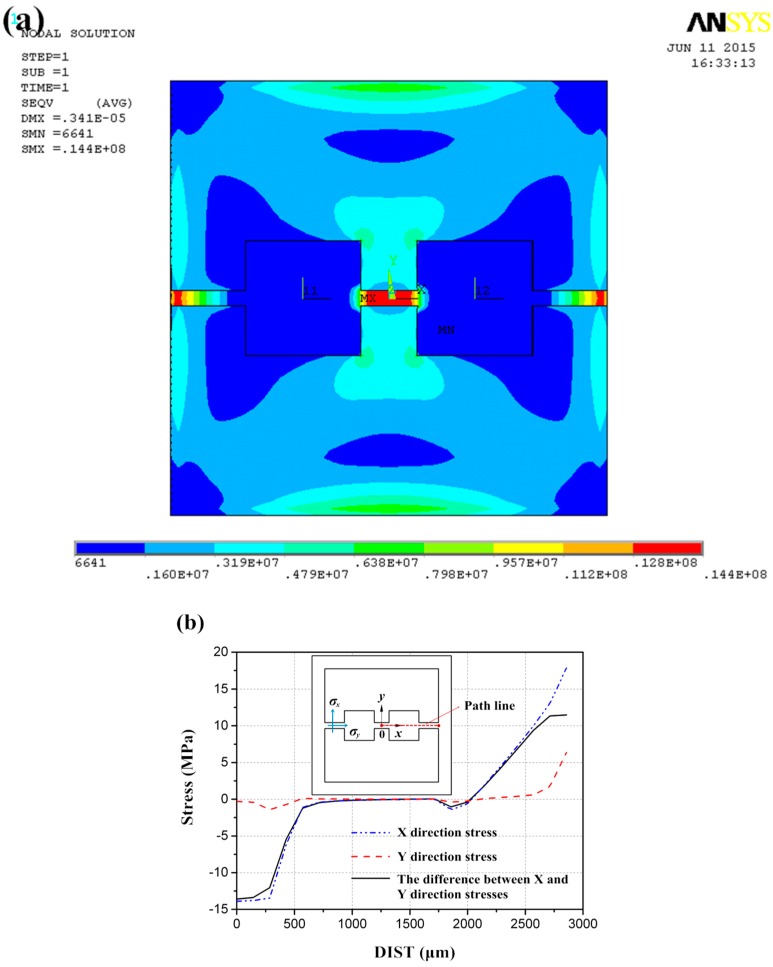
(**a**) The stress distribution of proposed structure; (**b**) The stress path along x-axis.

The numerical simulation program ANSYS is employed to evaluate the performance of the proposed structure with the above dimensions. The concentration is taken to the distribution of von Mises stress and the stress path along x-axis from center to edge, which influences the location of resistors and the sensitivity respectively. As Figure 5 shows, the stress concentration appears in the red regions, where the resistors should be placed. Furthermore, the nonlinear regions of stress can be found at the edges of beams, where the piezoresistors should not be located.

**Figure 6:**
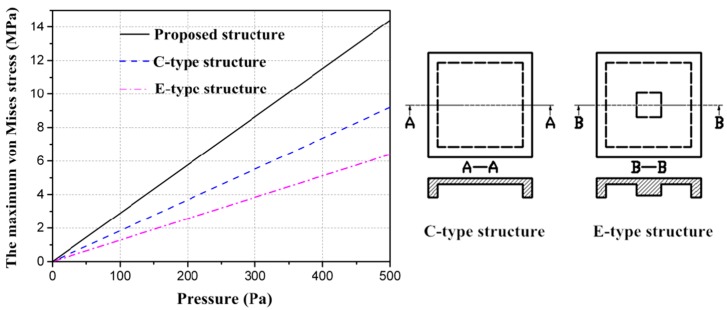
Acomparison of three typesof structures.

To compare the proposed structure with the C-type structure and E-type structure, which have been widely applied in various fields for measuring the micro-pressure of gas or water [1,2,15,16,17], all of them were simulated and the corresponding performances are plotted in Figure 6. Apparently, the proposed structure presents the highest sensitivity for the same dimensions. Furthermore, the stiffness is increased as a result of the introduction of beams and islands, so deflection is decreased and better linearity can be expected.

In order to theoretically estimate the specific value of the sensitivity of the new structure, the output voltage equation is given (take the resistor oriented in <110> direction on a (100) *n*-type silicon wafer) [18,19]:

(5) $$U_{o}(p)\approx \frac{1}{2}\pi_{44}(\sigma_{x}-\sigma_{y})U_{i}$$

where *U_o_(p)* and *U_i_* are the output and the input voltages, π_44_ is the shearing pirezoresistance coefficient, *p* is the applied pressure, and *σ_x_* and *σ_y_* are the longitudinal and transversal surface stresses at the central point of the resistor as labeled in Figure 5.

Consideration is given to temperature features as well as sensitivity, and the ion implantation concentration is set as 3 × 10^14^ cm^−^^3^ less than 1 × 10^17^ cm^−^^3^, so π_44_ can be set as 138 × 10^−7^ cm^2^/N. Furthermore, the difference between *σ_x_* and *σ_y_* has been calculated by ANSYS ranging from applied pressure *p*, so output voltage *U_o_(p)* is derived from Equation (5):

(6) $$U_{o}(p)\approx 0.414\times 10^{-5}{\left(\sigma_{x}-\sigma_{y}\right)}_{500\mathrm{Pa}}$$

where the sensitivity of 0.016 mV/V/Pa can be deduced.

### 2.3. Fabrication

The microchip adopted in this sensor is fabricated based on the bulk micromachining of a standard double side polished *n*-type (100) silicon wafer, whose thickness is 380 μm, and *p*-type piezoresistors are doped. *P*-type doping is adopted for two reasons: first, the piezoresistive coefficient of single crystal silicon is determined by the crystal orientations and doping types. To achieve the highest sensitivity on a (100) silicon layer, *p*-type resistors can be arranged at the edge of beams along with <110> direction, while *n*-type resistors have to be rotated 45° [20]. Second, heavily doped p^+^-Si is less influenced by temperature than heavily doped n^+^-Si.

**Figure 7:**
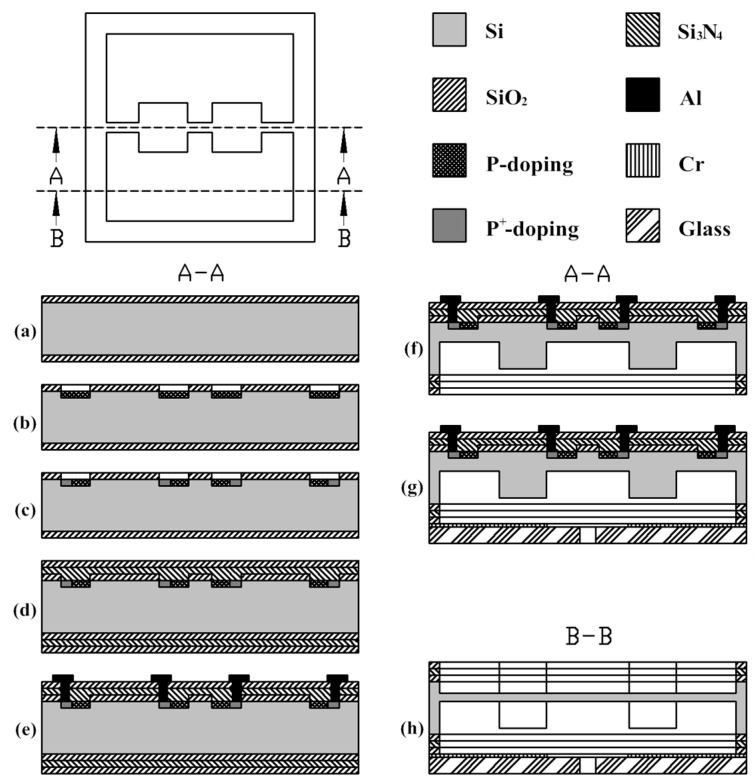
Fabrication process of the proposed chip.

**Figure 8:**
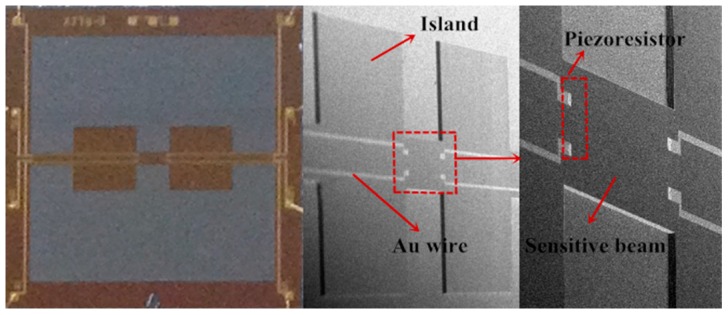
The micrograph of the finished sensor chip.

The detailed fabrication stepsare illustrated in Figure 7. Photolithography is employed and a total of six masks are need, five for the sensing element and one for the metal layer on the glass wafer [21]. (a) At first, SiO_2_ layers are grown on both sides of the substrate by thermal oxidation; (b) then, the inductively coupled plasma (ICP) techniqueis used to etch the damping gap on the front side of the silicon wafer after which implantation of boron is carried out with a concentration of 3 × 10^14^ cm^−3^ approximately forming a sheet resistance of 100 Ω; (c) heavy boron ion diffusion follows to create the connection between Al and the piezoresistors; (d) after, the passivation layers of Si_3_N_4_ and SiO_2_ are deposited and patterned by low pressure chemical vapor deposition (LPCVD) and plasma enhanced chemical vapor deposition; (e) Then contacts are created through photo patterning and etching technology on the front side utilizing reactive ion etching. The annealing technology should be executed at 1100 °C for 30 min under nitrogen in order to activate the boron ion electrically and make the dopant uniform. To connect resistors and wire through the formation of bonding pads, a metallization process is performed to sputter Al. Furthermore, a sintering process is involved to strengthen the ohmic contacts between Al wires and piezoresistors; (f) then to create a cavity and simultaneously avoid forming a bevel generated by KOH etching which is unfavourable for miniaturization, inductively coupled plasma etching is used on the back side of the wafer after patterning; (g) finally, the back side of the wafer is attached to Pyrex 7740 glass, with an anti-adsorption electrode made of Cr sputtered on the glass, by anodic bonding process. A microphotograph of the finished sensor chip is shown in Figure 8.

### 2.4. Package and Measurement

**Figure 9:**
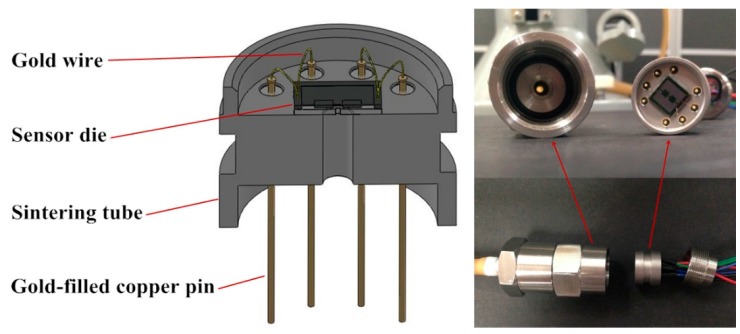
The schematic diagram and photos of the package.

**Figure 10:**
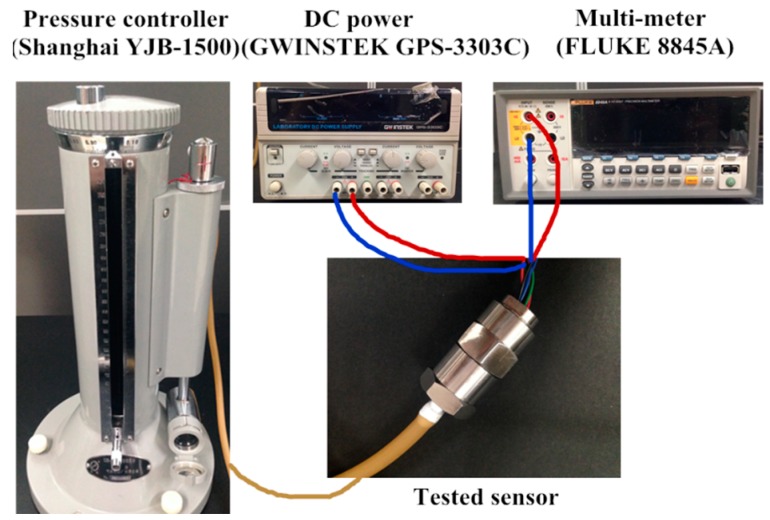
Static experimental setup for testing.

**Figure 11:**
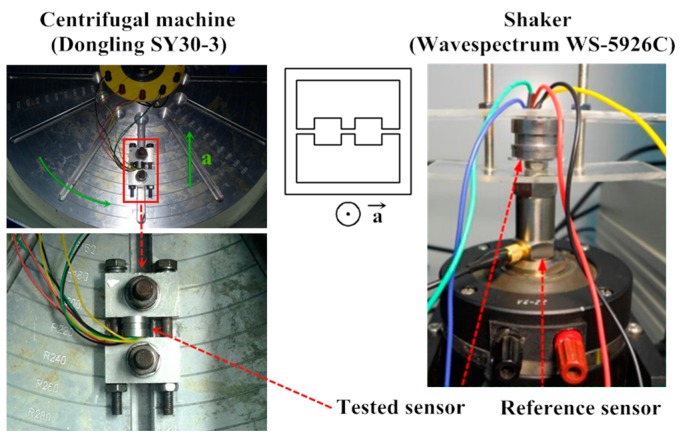
The acceleration and modal calibration setup.

The sensor is simply packaged for characterization as shown in Figure 9. The whole sensor chip is adhered to a stainless steel shell. The electrical connections between the pads in sensor chip and gold-filled copper pins are realized by gold wires. The static experimental setup is established as shown in Figure 10 and the static performance of the developed sensor is tested. The tested sensor is mounted into the outer tube connected to a compressor and pressure monitor through the rigid transfer frame. The compressor is used to control the pressure applied on the sensor chip, simultaneously monitored by the pressure meter to calibrate the tested sensor, whose Wheatstone bridge is excited with a 3 V DC power supply. The dynamic characteristics of the proposed sensor are tested with a set of calibration systems shown in Figure 11. A stable centrifugal machine is used for acceleration calibration along the normal direction of the membrane of the sensor. The natural frequency is calibrated though fixing the tested sensor and a reference sensor on the mechanical shaker. A peak concerning the voltage ratio will be generated when a sine sweep frequency passes through.

## 3. Results and Discussion

### 3.1. Experimental Results

**Figure 12:**
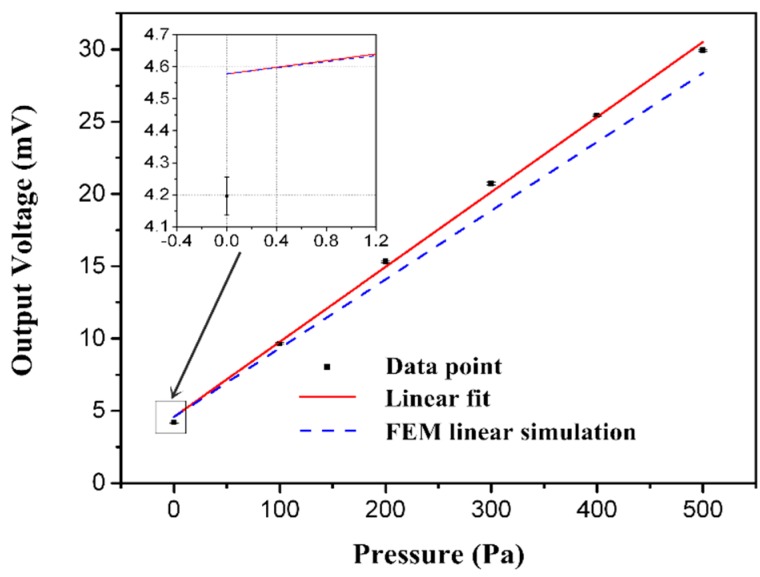
The output voltage of sensor.

**Figure 13:**
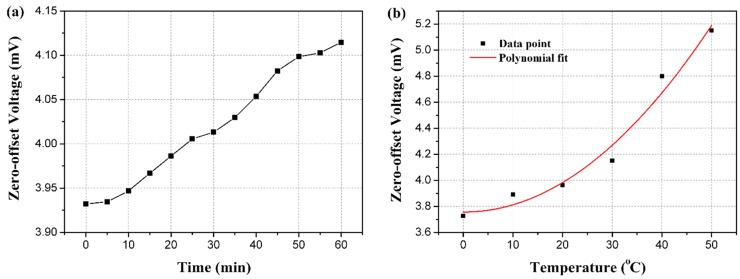
(**a**) Time-drift of the proposed sensor; (**b**) Temperature-drift of the proposed sensor.

Figure 12 shows the unamplified output voltage versus the pressure at room temperature. Meanwhile, the linear simulation result is depicted for comparison. The measured sensitivity of the tested sensor is 17.339 μV/V/Pa with a maximum non-linearity of 2.556%FS. The time-drift and temperature-drift of the proposed sensor are respectively measured within the temperature range from 0 °C to 50 °C with a 10 °C interval and for 60 min. Figure 13 shows outputs versus temperature and time.

**Figure 14:**
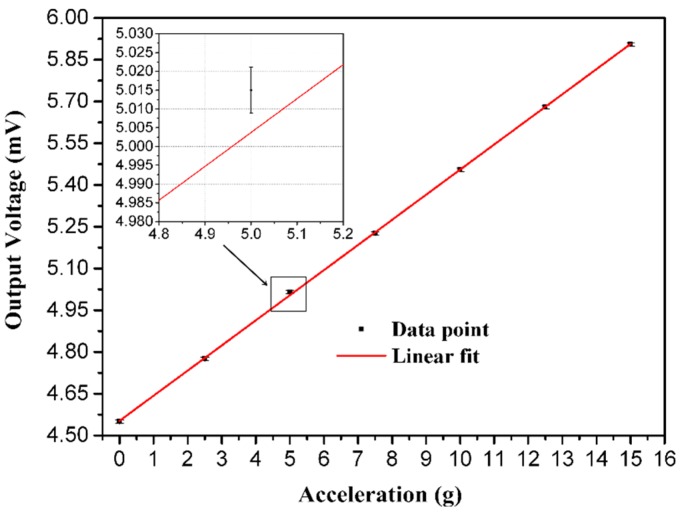
The influence of vibration on the proposed sensor.

**Figure 15:**
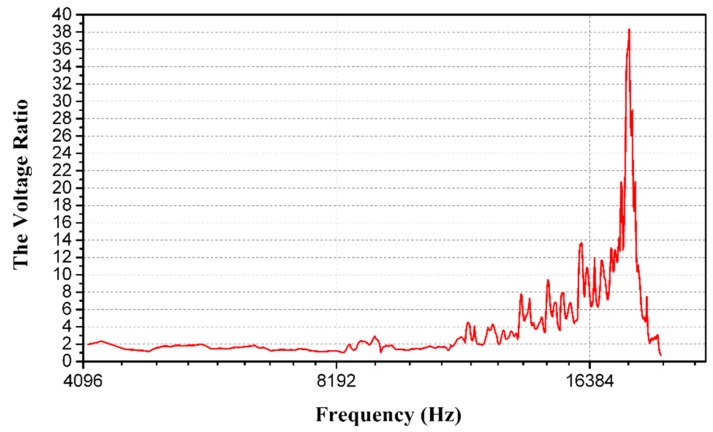
The resonant frequency of the proposed sensor.

The acceleration calibration result is drawn in Figure 14, in which the acceleration up 15 g with an interval of 2.5 g is imposed. The measurement result is described by the least squares method as well, and the maximum standard error of the testing point is enlarged. In the modal calibration, the peak induced by the resonance is drawn in Figure 15. By observing the peak of the curve, the resonant frequency of the proposed sensor could be figured out as 18.25 kHz.

The static characteristics are calculated on the basis of least square fitting results and listed in Table 2, demonstrating agreement between the full scale output voltage of actual measurement and the ANSYS calculated one with a deviation of 5.9%.

### 3.2. Discussion

The proposed sensor demonstrates the capability of high overload of 200 times overpressure and measurement with high sensitivity of 17.339 μV/V/Pa as Table 3 shows. The sensitivity in this work is more than nine times that reported in [1] and the overload resistance is enhanced by nearly 5%. In contrast with the sensors established in [2], which feature a high sensitivity of 12 μV/V/Pa, the sensitivity of the proposed sensors 1.44 times that, and the overload resistance is 8.65 times higher. Additionally, as bulk silicon micromachining technology has been utilized, low cost and mass production have been achieved, unlike [2] where SOI has been used to produce ultrathin film to provide high sensitivity.

Apparently, there is a deviation of 5.9% between the simulated and experimental results. The reason mainly lies in the calibration apparatus available in the laboratory, which features a precision of 42 Pa in 500 Pa pressure ranges equal to 8.4% FS. Due to the limited calibration accuracy, the precisions of the testing sensor and the equipment are of the same order. A ware of the finite accuracy, careful calibration of five pressure cycles has been done, which makes a small contribution to reducing the measurement errors. Besides, the residual stresses on Si_3_N_4_ and SiO_2_ passivation layers may also contribute to the relatively poor accuracy.

Zero-offset voltage is closely related to time and temperature as shown in Figure 13, which results from the different temperature coefficients of the four piezoresistors in the Wheatstone bridge due to the inconsistent dopant concentration of the resistors involved in the fabrication process step (b). The change of zero-offset voltage over time within 60 min is 0.38 mV (0.016% FS), although the zero point offset is slightly large, which relates to the precision of ICP technique involved in the fabrication process (b) as well as additional piezoresistive effect created by thermal stress produced during the packaging. The temperature coefficient of the offset output (TCO) of the proposed sensor can be calculated as 0.00135/°C within the temperature range from 0 °C to 50 °C with a 10 °C interval and 60 min stability time.

## 4. Conclusions

This work demonstrates a highly sensitive and overloading pressure sensor with a beam-membrane-dual-island structure. To verify the feasibility of the scheme, a prototype has been simulated, optimized, and fabricated. Test results reveal the effectiveness of the proposed structure to improve the sensitivity and the capability of bearing overload. Namely, the sensor features high sensitivity and overload capacity, and is promising for applications in the aviation field. Future work will be devoted to further studying the static performance of this device by improving the experimental conditions and the fabrication precision.

## Figures and Tables

**Table 1:** The parameters of the proposed sensor chip.

Parameter	*B*	*H*	*I*	*L*	*W*	*t*
Dimension (μm)	20–50	20–30	1500–1600	5400–5700	200–500	150–500

**Table 2:** Static performance of the proposed sensor.

Parameter	Value
Temperature (°C)	20
Supply voltage (V)	3
Zero point offset (mV)	4.196
Full scale span (mV)	23.781
Sensitivity (μV/V/Pa)	17.339
Nonlinearity (%FS)	2.556
Hysteresis (%FS)	0.514
Repeatability (%FS)	0.759
Accuracy (%FS)	2.715

**Table 3:** Comparison with other pressure sensors.

Sensor	Sensitivity (μV/V/Pa)	Overload Resistance (kPa)
Proposed sensor	17.339	100
Sensor in [1]	1.549	95
Sensor in [2]	12	10.36
Sensor in [13]	0.699	97.86
